# Mucin adhesion of serial cystic fibrosis airways *Pseudomonas aeruginosa* isolates

**DOI:** 10.3389/fcimb.2024.1448104

**Published:** 2024-08-22

**Authors:** Christian Herrmann, Meike Lingner, Susanne Herrmann, Inka Brockhausen, Burkhard Tümmler

**Affiliations:** ^1^ Institut für Biophysikalische Chemie, Medizinische Hochschule Hannover, Hannover, Germany; ^2^ Klinik für Pädiatrische Pneumologie, Allergologie und Neonatologie, Medizinische Hochschule Hannover, Hannover, Germany; ^3^ Department of Biomedical and Molecular Sciences, Queen’s University, Kingston, ON, Canada; ^4^ Biomedical Research in Endstage and Obstructive Lung Disease (BREATH), German Center for Lung Research, Hannover, Germany

**Keywords:** airway mucin, bacterial adhesion, cystic fibrosis, *Pseudomonas aeruginosa*, submaxillary mucin

## Abstract

The chronic airway infections with *Pseudomonas aeruginosa* are the major co-morbidity in people with cystic fibrosis (CF). Within CF lungs, *P. aeruginosa* persists in the conducting airways together with human mucins as the most abundant structural component of its microenvironment. We investigated the adhesion of 41 serial CF airway *P. aeruginosa* isolates to airway mucin preparations from CF sputa. Mucins and bacteria were retrieved from five modulator-naïve patients with advanced CF lung disease. The *P. aeruginosa* isolates from CF airways and non-CF reference strains showed a strain-specific signature in their adhesion to ovine, porcine and bovine submaxillary mucins and CF airway mucins ranging from no or low to moderate and strong binding. Serial CF clonal isolates and colony morphotypes from the same sputum sample were as heterogeneous in their affinity to mucin as representatives of other clones thus making ‘mucin binding’ one of the most variable intraclonal phenotypic traits of *P. aeruginosa* known to date. Most *P. aeruginosa* CF airway isolates did not adhere more strongly to CF airway mucins than to plastic surfaces. The strong binders, however, exhibited a strain-specific affinity gradient to *O*-glycans, CF airway and mammalian submaxillary mucins.

## 1 Introduction

Cystic fibrosis (CF) is an autosomal recessive trait that is caused by mutations in the *Cystic Fibrosis Transmembrane Conductance Regulator (CFTR)* gene (Grasemann and Ratjen, 2023). CFTR is a cAMP- and ATP regulated ion channel that conducts chloride and bicarbonate across the apical epithelial membrane (Lei et al., 2023). CF is a multi-system disorder of exocrine glands affecting the respiratory, gastrointestinal, hepatobiliary and reproductive tracts whereby the chronic microbial airway infections, particularly with *Staphylococcus aureus*, *Burkholderia cepacia* complex and *Pseudomonas aeruginosa*, are the major morbidity for most people with CF (Blanchard and Waters, 2019; Grasemann and Ratjen, 2023). *P. aeruginosa* may be eradicated by inhalative or systemic antimicrobial therapy during the early colonization phase (Parkins et al., 2018; Thornton and Parkins, 2023; Southern et al., 2024). However, later on it is virtually impossible to eliminate *P. aeruginosa*, even if CFTR modulators are applied that partially reverse the underlying basic defect in CF airways of perturbed ion and water flow (Middleton et al., 2019; Lopez et al., 2023) and reduce the bacterial load (Nichols et al., 2023; Armbruster et al., 2024; Dittrich et al., 2024).

The opportunistic pathogen *P. aeruginosa* thrives in aquatic habitats and can colonize the animate surfaces of plants, animals and humans. The global *P. aeruginosa* population consists of thousands of clones. However, the 25 most frequent clones have an absolute share of about 50% in the population (Wiehlmann et al., 2007, 2015). Besides some generalists such as clones C (ST17) and PA14 (ST253) that are found with similar frequency in disease and in the inanimate environment, major disease-associated lineages such as 1BAE (ST155) and 0C2E (ST395) and rare niche specialists colonize the lungs of people with CF (Rosenboom et al., 2023; Weimann et al., 2024).

When *P. aeruginosa* has chronically colonized the CF lungs, it typically will acquire a sessile lifestyle and persist in the bronchial lumen within microcolonies mainly made up of the mucus of the CF host (Worlitzsch et al., 2002; Ventre et al., 2006; Cramer et al., 2023). The mucus of a healthy person consists of 97% water and 3% solid (mucins, water, DNA, lipids, ions, proteins, cells and cellular debris), with the gel-forming mucins being the major structural component of mucus (Ermund et al., 2018a). CF is a muco-obstructive lung disease. Mucus hypersecretion and mucus plugging of the airways are the leading symptoms of a modulator-naïve patient with CF (Henderson et al., 2014).

Airway mucins are characterized by a high frequency of the hydroxy-amino acids serine and threonine, along with proline (PTS domain) and are glycosylated predominantly by the *O*-glycans, which typically make up more than 80% of the mass of a mucin. These central *O*-glycosylated mucin repeat domains are often interrupted by 100-amino-acid-long non-glycosylated CysD knot domains. Human airway secretions contain the mucins MUC4, MUC1, MUC16, MUC7, MUC5AC and MUC5B, whereby the latter two mucins are most abundant (Bechtella et al., 2024). Compared to respiratory secretions from healthy people, CF sputum has an increased level of MUC5AC and a decreased level of MUC5B (Henderson et al., 2014; Schaupp et al., 2023). The gel-forming MUC5AC and MUC5B form linear N-terminal covalent dimers whereby MUC5AC dimers moreover can non-covalently aggregate into net-like structures (Hansson, 2020).

The mucin-type *O*-glycans are extended from a common GalNAc bound to the glycoprotein through the hydroxyl group of a Ser or Thr side chain. There are four common *O* –GalNAc core structures in human respiratory mucins, i.e. core 1 (Galß1-3GalNAc), core 2 (GlcNAcß1-6(Galß1-3)GalNAc), core 3 (GlcNAcß1-3GalNAc) and core 4 (GlcNAcß1-6(GlcNAcß1-3)GalNAc) (Brockhausen et al., 2022). Comparing the *O*-glycosylation profiles of airway mucins in health and cystic fibrosis, CF mucins are less diverse in glycan structures, contain less core 3 and core 4 structures and are decorated to a higher degree with sialic acid, whereas fucosylation and sulfation are decreased (Davril et al., 1999; Xia et al., 2005; Schulz et al., 2007; Hayes et al., 2012; Bechtella et al., 2024).

Human airways are continuously exposed to microbes. Thanks to the mucociliary escalator, bundles of MUC5B polymers clean the tracheobronchial surface and remove bacteria in healthy airways (Roy et al., 2014; Ermund et al., 2017; 2018b). Conversely, MUC5AC becomes more abundant in CF airways and together with MUC5B can organize a stratified mucus layer that can separate bacteria from the epithelial surface (Hansson, 2020; Bos et al., 2023). Within CF lungs *P. aeruginosa* persists in the conducting airways together with human mucins as the most abundant structural component of its microenvironment. Hence, it is reasonable to assume that *P. aeruginosa* adheres to mucin in order to sustain its sessile lifestyle in the microcolony. In line with this argument, *P. aeruginosa* has been shown to adhere to purified tracheobronchial mucin (Vishwanath and Ramphal, 1984; Ramphal et al., 1991). Since only one of the eight test strains was of CF airway origin and the tracheobronchial mucin was from a non-CF donor, the issue of a habitat-specific adaptation of *P. aeruginosa* to mucin binding could not be addressed (Vishwanath and Ramphal, 1984; Ramphal et al., 1991). Here we report on the adhesion of serial CF airway isolates to airway mucin preparations from CF sputa. Mucins and bacteria were retrieved from modulator-naïve patients with advanced CF lung disease.

## 2 Material and methods

### 2.1 Patients and collection of sputa

Sputum was collected from seven hospitalized people with CF with advanced lung disease during their morning session of autogenic drainage performed under the supervision of the physiotherapist. Sputum was retrieved from five *P. aeruginosa*-positive CF patients (**Table 1**) and two *P. aeruginosa* – negative CF patients (patient 6, male, 29.0 years; patient 7, male, 13.5 years). Samples were stored at -20°C until use. Patient 1 has been seen at the outpatient CF clinic of Hannover Medical School since age of diagnosis, whereas all other patients had been referred from their local doctors to Hannover Medical School because of their advanced lung disease. Clinical data were extracted from the original paper medical records. Written informed consent was obtained from all study participants and their parents. In particular, patients provided informed consent to have their sputum used for research purposes. The study has been approved by the Ethics Committee of Hannover Medical School (study no. 3739).

**Table 1:** Patients’ characteristics.

Patient No.	1	2	3	4	5
Gender/pancreatic status*	male / PI	male / PI	male / PI	male / PI	male / PI
*CFTR* genotype	F508del/F508del	F508del/F508del	F508del/R347P	F508del/F508del	F508del/F508del
Age at diagnosis [yrs]	3.4	1.1	10.2	4.8	2.3
Age at onset of chronic	15.5	2.4/11.8^§^	17.7	18.6	9.2
*P. aeruginosa* colonization [yrs]					
*P. aeruginosa* clone type^$^	1BAE	D421	6D92	E84A	0C2E
Range during collection period					
Age	16.2 – 17.4	12.6-14.8	19.7-22.0	21.8-23.9	19.1-21.0
Crispin Norman Score	19	15	22	25	19
FEV1 [% pred.]	46-55	42-59	15-20	18-25	14-29
BMI [kg/m^2^]	21.2-21.4	13.8-14.6	13.2-15.7	16.0-17.4	16.8-18.7

*PI, exocrine pancreatic insufficient.

^$^Hexadecimal code according to Wiehlmann et al. (2007).

^§^Onset of colonization with clone D421.

### 2.2 Bacteria

Deep throat swabs or sputa were collected on three to four occasions over a 2-year period from the five *P. aeruginosa* – positive people with CF (**Table 1**). Swabs or sputa were inoculated onto Columbia blood and McConkey agar plates without antibiotics. The plates were screened for *P. aeruginosa* strains after 24 and 48 hours of incubation at 37°C. *P. aeruginosa* isolates were identified by colony morphology, growth at 42°C, characteristic pigment production, and the metabolism profile in the API 20 NE system (bioMérieux, Nürtingen, Germany). The taxonomic assignment of a *P. aeruginosa* strain was validated by MALDI-TOF mass spectrometry (Axima-Assurance-Shimadzu/SARAMIS-*AnagnosTec*, Shimadzu Deutschland, Duisburg, Germany). Secondary subcultures of isolates of different colony morphology were stored in soy tryptone broth supplemented with 17% (vol/vol) glycerol at -80°C until use. Strain typing was performed by a custom-based microarray (Wiehlmann et al., 2007), DraI or SpeI macrorestriction fragment pattern analysis (Römling et al., 1994), pyocin typing (Fyfe et al., 1984) and/or phage typing with the set of 21 phages described by Asheshov (1974). The genomes of strains TBCF10839, TBCF121838 and clone 1BAE strains were sequenced on SOLiD5500XL, Illumina and/or PacBio platforms as described previously (Klockgether et al., 2013; 2018; Wiehlmann et al., 2023) (Sequence Read Archive (SRA) of the EBI: study Accession No. ERP001300; Bioproject at NCBI: PRJNA975170; accession No. at NCBI: CP127016).

Besides the serial CF isolates, non-CF *P. aeruginosa* reference strains were included into the study on mucin adhesion, i.e. the O serotypes O4 (ATCC 33351), O6 (ATCC 33353), O9 (ATCC 33356) of the International Antigenic Typing System (IATS) (Liu and Wang, 1990; Moustafa et al., 2023) and strain PAO1 (DSM 1707, serotype O5), the latter being the major strain used for studies on *P. aeruginosa* biology (Brinkman et al., 2021).

### 2.3 Isolation and characterization of mucins from CF sputa

Mucins were isolated from sputum of the six CF patients 2 – 7 as described by Snyder et al. (1982) with modifications. The gentle procedure consists of multiple centrifugation, dialysis and lyophilization steps so that the composition in sputum and the native structure of the polydisperse highly glycosylated mucins (mol. wt. > 10^6^ Da) are retained. To cope with the changes of ionic strength and pH and the increase of volumes during each step, repeated lyophilizations are necessary. First, cellular debris is removed by centrifugation. Next, low molecular weight compounds and ions are removed by dialysis in the presence of EDTA in unbuffered solution. After lyophilization, the ionic interactions between the negatively charged sialic acid and sulfate of the mucins with the amino groups of non-covalently bound sputum proteins are eliminated. The ϵ-amino groups of the lysine residues of the non-glycosylated proteins are neutralized with citraconic anhydride in Tris buffer at pH 8.0. After lyophilization, the mucins are precipitated in unbuffered solution at pH 4.8 with the detergent N-Cetyl-N,N,N trimethyl ammonium bromide. After anew dialysis and lyophilization a mixture of Coomassie-blue stain-negative, highly glycosylated, high mol.wt. mucins is obtained.

Procedure: Sixteen ml sputum were homogenized with 80 ml ice-cold isotonic saline in a beaker by multiple aspirations with a 10 ml sterile plastic syringe (0.9 x 40 mm cannula). After dropwise addition of 1 ml 1%-sodium azide (Sigma-Aldrich) to inactivate microbes, the homogenate was stirred (10 – 20 rpm) (Heidolph Hei-Mix S) at 4°C for 24 h. After centrifugation at 2,000 g for 10 min, the supernatant was collected. The extraction was continued by two cycles of washes of the pellet with 50 ml ice-cold isotonic saline each, centrifugation of the homogenate at 2,000 g for 10 min and the collection of the supernatant. The supernatants were combined and after the addition of 0.1 M EDTA to a final concentration of 1 mM EDTA were dialyzed (VISKING dialysis tubing, MWCO 12000) for in total 72 h at 4°C against two changes of 0.01 M NaCl and four changes of bidistilled water. After freeze-drying, the lyophilisate was dissolved in 40 ml 0.5 M Tris-HCl, pH 8.0. Twenty drops of citraconic anhydride (Sigma-Aldrich) were added slowly under constant stirring. The solution was dialyzed for in total 24 h at 4°C against two changes of bidistilled water and then freeze-dried. The dry lyophilizate was dissolved in 20 ml bidistilled water and adjusted to pH 4.8 under stirring with acetic acid. The mucin was precipitated by slowly adding dropwise a total of 0.5 ml 20% (w/v) N-Cetyl-N,N,N trimethyl ammonium bromide (GR for analysis, Sigma-Aldrich). The solution was kept for 12 h at 4°C and then centrifuged at 50,000 g for 30 min at 4°C. The sedimented mucin was dissolved in a small volume (10 – 15 mL) of 0.5 M NaCl and dialyzed for in total 24 h at 4°C against two changes of bidistilled water. The dialysate was diluted to 150 mL with 0.5 M NaCl, dialyzed for in total 24 h at 4°C against two changes of bidistilled water and then freeze-dried. The yield of the lyophilizate was determined with an analytical balance (± 0.1 mg).

The mucin preparations were characterized by Coomassie stain of the separating gel of 10% SDS-PAGE and the profile of Sepharose 2B-CL (Cytiva) gel elution chromatography. Fractions of the eluate were examined for their sugar content by spectrophotometry of the reaction with phenol/concentrated sulfuric acid (DuBois et al., 1956). The monosaccharide composition of the CF mucin preparations was determined by gas chromatography. One mg mucin preparation was dissolved in 2 ml CH_3_OH. After the addition of 2 ml 0.5 M HCl/CH_3_OH, the solution was incubated at 70°C for 4 h. The solution was evaporated, and the residue was dissolved in 100 µL pyridine. After a further evaporation, 100 µL pyridine, 50 µg of the standard arabitol and 100 µL N,O-bis(trimethylsilyl)-trifluoroacetamide were added. After a 1 h incubation at 70°C, 0.5 µL of sample and 1 µL of reference (C_12_ – C_20_ alkanes, 50 µg/ml) were injected into the gas chromatograph with a flame ionization detector (injection and detection at 300°C). Compounds were separated at a constant temperature of 104°C on 20 m home-built glass capillaries with hydrogen as carrier gas.

### 2.4 Submaxillary mucins and synthetic oligosaccharides

**Figure 1:**
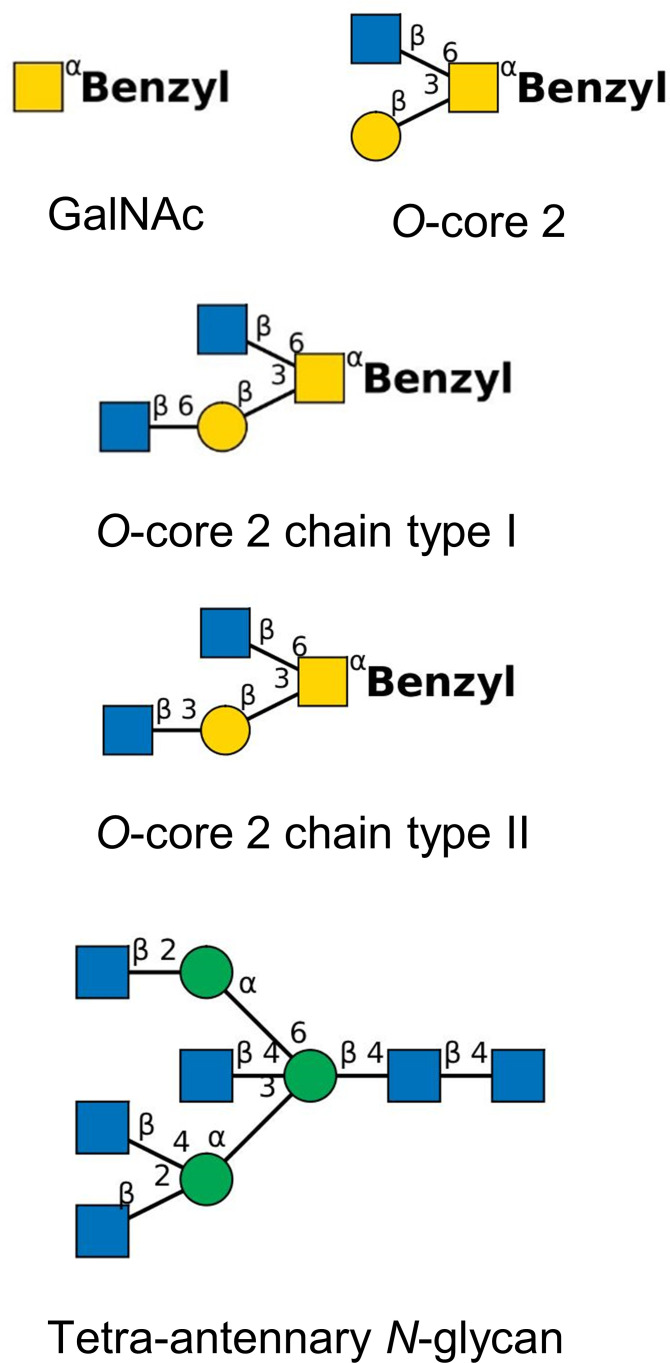
Synthetic tetra-antennary *N* –glycan and benzylated *O*-glycan structures used for competition experiments (yellow square, *N*-acetylgalactosamine; yellow circle, galactose; blue square, *N-*acetylglucosamine; green circle, mannose). Structures have been drawn with the software tool ‘DrawGlycan-SNFG’ (Cheng et al., 2017).

CF airway mucins are decorated with dozens of different extensions of core 1 and core 2 structures (Bechtella et al., 2024). To identify any structural selectivity of bacterial adhesion to *O*-glycans, we included synthetic oligosaccharides with core 2 structures (**Figure 1**) and submaxillary mucins that are decorated with a less complex core 1 *O*–glycan repertoire into our mucin adhesion studies. Ovine submaxillary mucin (OSM) (Konstantinidi et al., 2022) only carries (NeuAcα2–6)GalNAcα1 in its highly glycosylated tandem PTS repeats. Porcine submaxillary mucins (PSM) (Gerken et al., 2002) are decorated with up to 16 different extensions of core 1 structures. Conversely, bovine submaxillary mucin (BSM) (Vos et al., 2023) carries a highly diverse repertoire of hundreds of *O*-glycans. To reduce the complexity of the *O*– glycan repertoire of OSM and PSM even further, we prepared asialo-OSM and asialo-PSM (Yang et al., 1994). OSM or PSM was treated with 0.1 N H_2_SO_4_ for 1 h at 80°C to remove sialic acid followed by dialysis and lyophilization. Next, the mucin (250 mg) was incubated for 24 h at 37°C with 0.5 U bovine kidney α-fucosidase (New England Biolabs), 0.2 U chicken liver α-fucosidase (Oxford GlycoSystems) and only in case of OSM in addition with 1.5 U bovine testicular β-P-galactosidase (kindly donated by Dr W. Jourdian, University of Michigan, Ann Arbor, MI) in 25 ml 100 mM Na-citrate-phosphate buffer (pH 5). The mixture was heated to 80°C for 10 min and mucin was purified on a Sephadex G25 column, equilibrated in water.

OSM and PSM were donated from the late Harry Schachter, Toronto. Fresh submaxillary glands were obtained at a slaughter house and kept frozen until used. PSM was prepared according to the procedure of Hashimoto et al. (1964) modified by De Salegui and Plonska (1969). OSM was prepared according to the procedure of Tettamanti and Pigman (1968) modified by Hill et al. (1977). The monosaccharide composition was determined by gas chromatography as described above. BSM was purchased from Sigma-Aldrich. Synthetic oligosaccharides were a gift from Khushi L. Matta, Buffalo, and Hans Paulsen, Hamburg. Oligosaccharide structures (**Figure 1**) are drawn in the format of the ‘Symbol Nomenclature for Glycans (SNFG)’ (https://www.ncbi.nlm.nih.gov/glycans/snfg.html, last update February 26, 2024) (Varki et al., 2015; Neelamegham et al., 2019).

### 2.5 Test: adhesion of *P. aeruginosa* to mucin

The adhesion assays were performed with sterile Nunc™ 96-well polystyrene U-bottom microplates (Thermo Fisher Scientific). 48 wells of the microplate were coated with 200 µl mucin solution (10 µg mucin/ml PBS) per well and the other 48 wells with 200 µl phosphate buffered saline (PBS) per well. Subsequently, the plate was kept at 37°C for up to 18 h until the adhesion assay was started.

For mucin adhesion assays, two to eight *P. aeruginosa* strains were processed in parallel. Small colony variants (Häussler et al., 1999) and multicellular aggregates (Kragh et al., 2016) were not included into the assay because in our hands these morphotypes did not allow a reliable determination of bacterial counts by optical density or plating. In accordance with our experience of fitness experiments that reproducibly produced planktonic communities at late exponential phase (Cramer et al., 2022; Kuschnerow et al., 2023), bacterial growth was standardized by protocol as follows: A loop of the *P. aeruginosa* isolate stored in the micro-tube was streaked onto a LB agar plate and incubated for up to 24 h at 37°C. The next day in the afternoon, 25 ml lysogenic broth (LB) in a 100 ml culture flask were inoculated with a colony and cultivated overnight at 150 rpm at 37°C. The next morning, fresh 25 ml LB medium was inoculated with 1 ml of the overnight culture. The bacterial suspension was grown at 150 rpm and 37°C for 5 h to late exponential phase. After spectrophotometric determination of the optical density of the culture at 600 nm an aliquot of 2 ml of the culture was diluted with PBS to OD_600_ of 0.6.

Each OD_600 =_ 0.6 bacterial suspension of the *P. aeruginosa* strains was then diluted ten-fold (suspension I) and hundred-fold (suspension II) with PBS using sterile 10 ml glass pipettes. To start the mucin adhesion assay, PBS was removed from the mucin- coated microplate by suction. Mucin-coated and un-coated wells of the microplate were inoculated each in triplicate with either 100 µl of the ten-fold (suspension I) or 100 µl of the hundred-fold dilution (suspension II) of the OD_600 =_ 0.6 suspension of one bacterial strain. The microplate and the diluted bacterial suspensions I and II of the strains were incubated for 30 min at 37°C. By the end of the 30 min incubation period, the diluted bacterial suspensions were stored at 4°C and the microplate was kept at room temperature. Fluid was removed from the wells of the microplate with a Pasteur pipet by suction. Each well was washed 10 times with 250 µl PBS. 300 µl 1% (vol/vol) Triton X 100 in PBS was added to each well and incubated for 30 min at room temperature to release mucin and bound bacteria. Thereafter CFUs were determined by plating tenfold dilutions of 100 µl aliquots of the Triton X100 suspensions and of the bacterial suspensions kept at 4°C, respectively.

In case of the competition experiments with oligosaccharides, two microplates were used per bacterial strain. Fifty µl of PBS were added to each well of rows 1 and 8 of the microplate. Fifty µl of oligosaccharide 1 in PBS were added to each well of rows 2 (0.5 µg/ml) and 3 (5 µg/ml). Correspondingly, solutions of oligosaccharides 2 and 3 were distributed to the wells of rows 4/5 and 6/7, respectively. Another PBS control and oligosaccharides 4 and 5 were distributed on the second microplate. Thereafter, the procedure matched that of the standard mucin adhesion assay described in the previous paragraph. Mucin-coated and un-coated wells were inoculated each in triplicate with 50 µl of five-fold or fifty-fold dilutions of the suspension of the bacterial strain, incubated at 37°C for 30 min and washed with PBS. Bound bacteria were released with detergent and quantified as CFUs.

All mucin adhesion assays were performed with three technical replicates on the microplate (see above) and two independent biological replicates.

### 2.6 Statistics

Since the Shapiro – Wilk test revealed that all datasets were not normally distributed, non-parametric tests were performed. First, the datasets of the adhesion of serial isolates to polystyrene and the CF mucin preparations displayed in **Table 2** were evaluated whether differential features of the mucin or differential features of the bacterial clonal variants were more important for the adhesion of bacteria to mucin. Mann-Whitney U Rank tests were performed on the adhesion of serial *P. aeruginosa* isolates to four targets, i.e. polystyrene and the three CF mucin preparations from CF donors 3, 4, and 5. Ranks were assigned for all pairwise comparisons of the ratio of bound CFU to total CFU. In dataset A all *n x (n-1)/2* pairwise comparisons of the ratios for the adhesion of all *n* colony morphotypes retrieved from one sputum sample (I) or one genotype (II) were pooled for the four targets. In dataset B the six pairwise comparisons of the ratios for the adhesion of one individual colony morphotype to four targets (polystyrene and the mucin preparations from CF patients 3, 4, 5) were pooled for each colony morphotype retrieved from one sputum sample (I) or one genotype (II). Datasets A and B were then compared by Mann Whitney rank test.

**Table 2:** Adhesion of serial *P. aeruginosa* CF airway isolates to polystyrene and CF mucin provided as the ratio of bound CFU to total CFU in percent*.

Patient No.	clone	*P. aeruginosa* colonization time [yrs]	Plastic polystyrene^#^	Mucin preparation from CF donor		
				3	4	5
1	1BAE	0.7	10; 4; 6	4; 2; 0.7	8; 3; 0.4	10; 7; 0.1
		1.3	78; 1; 2	36; 1; 20	21; 0.06; 17	25; 0.8; 5
		1.9	5; 15	1; 3	0.5; 2	0.5; 3
2	D421	10.2	0.3; 0.01	0.04; 0.04	0.4; 0.4	0; 0
		11.4	0.2; 0; 0.3; 0.02	0.3; 11; 12; 0.2	0.4; 17; 50; 0.4	0; 25; 40; 0.2
		11.7	0.05	1.5	1.5	1.5
		12.4	0.3; 0.2	0.1; 0.1	0.4; 0.3	0.1; 0.1
3	6D92	2.0	12; 0.01	6; 0.1	10; 0.1	6; 0.2
		3.0	13; 7	5; 3	6, 2	7; 2
		4.3	0.01; 15; 14; 0.05	0.03; 8; 5; 0.05	0.05; 7; 5; 0.04	0.03; 6; 5; 0.03
4	E84A	3.2	0	0.01	0.01	0.01
		4.4	9; 7	1, 1.5	0.5; 1.3	0.1; 0.7
		5.3	2; 0.05; 0.01	3; 0.05; 0.01	4; 0.03; 0.01	3; 0.04; 0.01
5	OC2E	9.9	40	50	18	60
		10.8	0.05; 1.5; 0.1; 30; 19; 0	0; 0.6; 0.2; 27; 23; 0	0; 0.9; 0.2; 16; 18; 0	0; 0.6; 0.2; 20; 30; 0
		11.8	0.2; 0.1	0.4; 0.1	2.5; 0.1	0.2; 0.1

*Values are the mean of three biological replicates each examined by three technical replicates. The isolates with different colony morphotypes retrieved from the same sputum sample are shown in the same order in their adhesion to plastic and to mucin preparations from CF donors 3, 4, and 5.

^#^uncoated well in microtiter plate.

The datasets shown in **Table 3** were evaluated by Kruskal Wallis tests followed by the *post-hoc* Dunn’s test using a Bonferroni corrected alpha value for all pairwise comparisons. Calculations were performed with online tools provided by Statistics Kingdom (https://www.statskingdom.com/).

**Table 3:** Adhesion of *P. aeruginosa* strains to CF mucins and submaxillary mucins provided as the ratio of bound CFU to total CFU in percent*.

Strain	plastic polystyrene^#^	Mucin preparations from sputum of CF donor						Submaxillary mucins					95% confidence interval of the median of adhesion to mucins
		2	3	4	5	6	7	OSM	a-OSM	PSM	a-PSM	BSM	
TBCF10839	3	70	64	7	58	25	77	49	70	115	98	98	34 - 100
	19	65	73	8	43	48	60	53	55	74	101	37	
CF1-7	4	80	31	36	35	9	25	23	36	7	63	59	16 - 38
	6	46	21	45	19	25	29	23	14	11	59	34	
CF1-8	20	51	31	48	38	60	36	32	63	69	88	44	31 - 60
	18	26	19	30	56	49	14	48	35	73	44	27	
CF1-10	49	94	21	86	99	52	51	72	58	60	102	79	61 - 95
	53	74	53	60	101	72	41	68	74	70	98	89	
CF2-7	0.1	2.4	0	3.4	1.2	0	1.7	0.1	0	0.1	0.1	0.1	0.3 – 0.5
	0	1.4	0.1	4.4	0.5	0	0.8	0.1	0	0.1	0	0.1	
CF2-8	1.1	4.0	1.7	2.0	1.4	1.4	6.1	0.3	0.3	2.6	2.3	0.2	0.0 – 2.6
	2.3	3.1	1.5	1.0	0.1	0.4	1.9	0.5	0	0	0	0.4	
CF3-7	17	5	47	19	65	39	14	37	25	33	33	7	12 - 47
	5	25	60	34	64	75	33	28	14	31	21	21	
CF4-4	28	7	8	5	9	12	4	8	6	14	6	6	7 - 11
	33	9	7	6	12	9	4	4	5	13	13	5	
CF5-8	19	25	7	23	4	16	10	17	23	22	21	27	18 - 24
	31	20	6	21	4	19	8	11	17	16	17	18	
CF5-9	48	42	6	25	4	39	30	20	27	53	41	35	29 - 50
	64	70	10	50	3	31	13	28	34	30	50	39	
PAO1	3	17	0	6	5	9	18	8	13	13	13	26	2 - 17
	3	12	2	4	6	4	13	4	6	5	4	7	
IATS-O4	13	25	7	13	0	22	17	9	30	21	18	24	8 - 35
	18	15	10	19	5	37	15	10	11	25	30	35	
IATS-O6	15	8	3	2	11	8	24	7	4	8	13	8	4 - 16
	3	13	3	4	2	13	11	12	2	3	13	11	
IATS-09	11	17	6	15	11	14	14	5	24	15	21	14	8 - 19
	17	16	5	16	7	10	9	5	6	10	12	8	

*Each value of the two biological replicates is the mean of three technical replicates.

^#^uncoated well in microtiter plate.

## 3 Results

### 3.1 Origin of *P. aeruginosa* CF airway isolates

Forty-one *P. aeruginosa* isolates (**Table 2**) were collected on three to four occasions over a 2-year period from five CFTR modulator-naïve adolescents and young adults who were suffering from advanced to end-stage CF lung disease (**Table 1**). To cover the diversity of the *P. aeruginosa* community within a sputum sample, single colonies of all divergent morphotypes grown from the first loop streaked onto a blood agar plate, were implemented in the strain collection. Thus, one to six different colony morphotypes were retrieved from individual sputum samples or throat swabs (**Table 2**). Each patient was chronically harboring a single clone (Wiehlmann et al., 2007). Patients 1, 2 and 5 were carrying the common clones 1BAE (ST155), D421 (ST253, ‘PA14’ clone) and 0C2E (ST395), respectively, which belong to the six most frequent clone types in the CF patient population (Wiehlmann et al., 2015). Conversely, patient 3 was colonized with the uncommon clone 6D92 and patient 4 was colonized with the rare clone E84A that is not represented by any isolate from another source in our current collection of more than 5,000 *P. aeruginosa* strains. The high mucin binder TBCF10839 was isolated from patient 2 when he was critically ill and was suffering from repeated pulmonary exacerbations (Tümmler et al., 1991; Klockgether et al., 2013). After the TB clone had been eradicated by high-dose antipseudomonal chemotherapy, patient 2 became colonized with the PA14 lineage. He remained affected by a poor lung function, but did not experience any pulmonary exacerbation during carriage of the PA14 clone.

### 3.2 Airway mucin preparations from CF sputum

To isolate the mucins, we followed the protocol by Snyder et al. (1982) that mainly consists of multiple centrifugation, dialysis and lyophilization steps (see chapter 2.3). Proteins non-covalently bound to airway mucin by ionic interaction are removed by reversible modification of lysine residues with citraconic anhydride so that the *O-*glycosylated mucin can be precipitated with cetyltrimethylammonium bromide. The final gel filtration with Sepharose CL-2B yielded high molecular weight mucin preparations in the void volume that showed no Coomassie stain-positive proteins in the resolving gel of 10% SDS-PAGE. The CF mucin preparations contained the expected monosaccharides of *O*-glycans, i.e. GalNaC, Gal, GlcNAc, Fuc and NeuAc. Mannose contributed with 1-4 mol% to the monosaccharide composition consistent with the presence of *N*-glycans in the N- and C- terminal regions of Muc5B and Muc5AC (Thornton et al., 2008). The analogous gas chromatographic analysis of the submaxillary mucins revealed the presence of NeuAc and GalNAc in the OSM sample and GalNAc, Gal, Fuc and NeuAc in the PSM sample. The absence of GlcNAc in the PSM sample is consistent with the literature that PSM exclusively carries core 1 *O*-glycans (Gerken et al., 2002).

### 3.3 Adhesion of *P. aeruginosa* to mammalian mucin: standardization of the assay

Based on the report by Vishwanath and Ramphal (1984), we set up a standardized assay to compare the binding of *P. aeruginosa* strains to mucin in the 96-well format of a polystyrene microtiter plate. The assay conditions were optimized with two clonal variants of known genome sequence, the low mucin binder *P. aeruginosa* TBCF121838 and the high mucin binder TBCF10839, the latter isolated from CF donor 2 (Tümmler et al., 1991; Klockgether et al., 2013; Wiehlmann et al., 2023) (**Supplementary Table S1**). The following parameters were varied: coating of wells with mucin, inoculum and culturing time of a bacterial strain, contact time of bacteria with mucin, removal of non-adhering bacteria and the release of bound bacteria from the well with nonionic detergent. Bacterial adhesion to mucin saturated if the well had been coated with 0.5 to 1 µg mucin and the bacteria had been exposed for at least 20 minutes to mucin. The minimal concentration to release the mucin-bound bacteria was found to be 0.5% Triton X-100. Applying the resulting optimized protocol, 10^5^ to 10^7^ bacteria grown to early stationary phase were exposed for 30 min in 0.1 ml PBS to a well coated with 2 µg mucin. After removal of free bacteria with 10 washing steps, the bound bacteria were released for 30 minutes with 0.3 ml 1% Triton X-100 and the CFUs were quantified in triplicate. All subsequent adhesion studies were then performed with 96-well plates of the same lot. Using the standardized procedure, the strong binding of the TBCF10839 to airway mucins was confirmed with mucin preparations prepared from the donor of this strain and patients 3, 4 and 5 (**Table 3**).

### 3.4 Adhesion of serial CF airway isolates to CF mucin preparations

Next, we tested the adhesion of the 41 serial *P. aeruginosa* isolates to mucin preparations from CF donors 3, 4, and 5 and to polystyrene of uncoated wells (positive control) by applying the standardized protocol in 96-well microtiter plates (**Table 2**; Excel File in the **Supplementary Table S6**). The 41 P*. aeruginosa* strains retrieved from the 16 CF sputa of the five CF donors 1 – 5 showed a broad range from no to almost 100% binding of supplied CFUs. An adhesion of more than 10% to polystyrene and/or CF mucins was observed for 13 strains. A higher binding to a CF mucin preparation than to polystyrene was detected for one 1BAE and five D421 strains. None of the 23 P*. aeruginosa* isolates retrieved from CF donors 3, 4 and 5 exhibited a higher adhesion to the mucin preparation from its CF lung habitat than to the mucin preparations from the other two CF hosts.

A more than 10-fold difference in bacterial binding between subclonal variants was found in seven of the 12 sputa carrying two or more discernable colony morphotypes. The between-strain variation of the adhesion to the same target was significantly higher than the within-strain variation of adhesion to polystyrene and the three mucin preparations (**Supplementary Table S3**). In other words, the adhesion of clonal variants to the same surface was more variable than the adhesion of a single strain to the four different surfaces (clone 1BAE, *P* = 0.01; D421, *P* = 0.02; 6D92, *P =* 0.0006; E84A, *P* < 0.00001; 0C2E, *P* < 0.00001; **Supplementary Table S3**). This finding told us that the affinity of *P. aeruginosa* clonal variants to mucins from CF sputum was more strongly determined by the differential intraclonal repertoire of bacterial adhesins than by the differential spectrum of epitopes in mucin preparations.

### 3.5 Adhesion of selected *P. aeruginosa* strains to submaxillary and airway mucins

**Figure 2:**
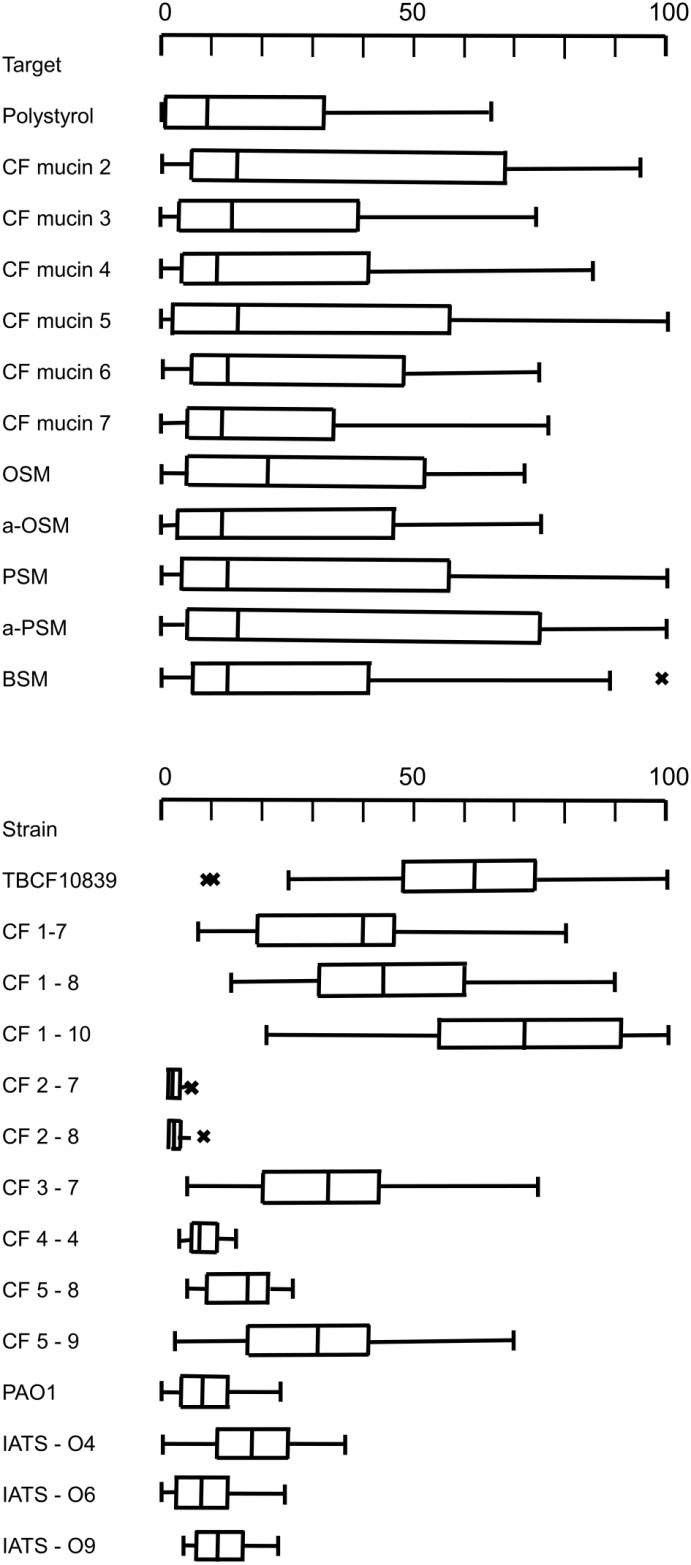
Upper panel: Boxplot presentation of the adhesion of 14 P*. aeruginosa* strains to polystyrene, the CF mucin preparations from sputa of CF donors 2, 3, 4, 5, 6, 7 and to the submaxillary mucins OSM, a-OSM, PSM, a-PSM and BSM. Lower panel: Boxplot presentation of the adhesion of *P. aeruginosa* strains TBCF10839, CF1-7, CF1-8, CF1-10, CF2-7, CF2-8, CF3-7, CF4-4, CF5-8, CF5-9, PAO, IATS-O4, IATS-O6, IATS-O9 to six CF mucin preparations and five submaxillary mucins. The center line of the boxplot depicts the median (50th percentile). The lower and upper boundary of the box represent the first (25th percentile) and third (75th percentile) quartile, and hence define the interquartile range (IQR). Whiskers extend from the box to the largest/smallest non-outlier data point (1.5 × IQR). Outliers are indicated by the symbol ‘x’.

The screening of the serial CF *P. aeruginosa* isolates identified a few strong mucin binders. We hypothesized that these strong binders should have acquired some specificity for the epitopes presented by CF mucin preparations, most likely carbohydrate moieties of the *O*-glycans of the PTS domains. Thus, besides reference strains of the common clones and serotypes we selected eight CF isolates that exhibited uniformly strong or disparate binding to the individual CF mucin preparations for a more refined analysis. Adhesion assays were performed with mucin preparations from sputa of six CF donors and five submaxillary mucins of divergent complexity of their repertoire of *O*–glycans (see section 2.4). The compilation of the same primary data in **Table 3** and in **Figure 2** shows some strain-, but no mucin-specificity of bacterial adherence. Each of the 11 mucins covered a broad range from weak to strong binding of the individual *P. aeruginosa* strains. The Kruskal-Wallis H test indicated that there was no significant difference of bacterial adhesion between the six CF mucin preparations, the five submaxillary mucins and polystyrene (*P* = 0.44, effect size: 0.00). Mucin-specific affinity for the binding of *P. aeruginosa* may exist but was overshadowed by the stronger strain-specificity of adhesion. Pairwise comparisons indicated non-significant trends of lower bacterial binding to polystyrene and to CF mucins 3 and 5 than to CF mucin 2 (uncorrected *P* values of 0.04; 0.02; 0.04) and to asialo-PSM (uncorrected *P* values of 0.01; 0.03; 0.04).

The adhesion profile was dominated by the differential affinity of the *P. aeruginosa* strains (*P* = 3 x 10^-12^; effect size 0.7). Whereas the four reference strains did not differ in their adhesion profile to the eleven mucins, the *Post-Hoc* Dunn’s test using a Bonferroni corrected alpha of 0.00055 indicated a significantly different binding of CF isolates to mucins and polystyrene for 43 of 85 pairwise comparison of strains (**Supplementary Table S4**).

The CF airway isolates exhibited a strain-specific profile of mucin binding (**Figure 2**). The two strains of the PA14 lineage were neither binding to polystyrene nor to mucins. A low median binding below 20% was observed for CF isolate 4-4 and a moderate binding was recognized for CF isolate 5-8. Six CF isolates were strong mucin binders, i.e. strains CF3-7, CF5-9, TBCF10839 and the clonal 1BAE variants CF1-7, CF1-8 and CF1-10 (**Figure 2**). Each strain of the six strong mucin binders showed an individual gradient of affinity for the mucin preparations, whereby no general preference for its CF host, the CF habitat and absence or presence of sialic acid was noted (**Supplementary Table S5**). The 1BAE variants CF1-7, CF1-8 and CF1-10 had been retrieved from the same sputum specimen. Their significantly different binding to the 11 mucins of our panel (*P* = 6x10^-7^, effect size 0.39) indicates that the CF lung may be colonized with a *P. aeruginosa* community of clonal variants with divergent mucin affinity profiles.

### 3.6 Competition of mucin and glycans for bacterial binding

The screening of the 14 P*. aeruginosa* isolates for their adhesion to the 11 mucins indicated that the strength and specificity of the interaction between mucin and *P. aeruginosa* is driven by the repertoire of binding sites of the bacterium. Next, we wanted to know whether *N*-glycan and *O-*glycan structures confer specificity to the bacterial mucin adhesion. Three *P. aeruginosa* strains, the two strongest mucin binders TBCF10839 and CF1-10 and the low binder IATS-O9, were examined in their adhesion to PSM and two CF airway mucins in the presence of variable amounts of neutral *O*- or *N*-glycans. The competition experiments were performed with the monosaccharide GalNAc, three type 2 *O*–glycans and a tetra-antennary *N*-glycan (**Figure 1**).

Results are shown in **Table 4**. The reader may note that the bacteria did not only bind to the oligosaccharide but also utilized them as the sole carbon source to grow in PBS as it is indicated by normalized values larger than 100% for several ternary combinations. The monosaccharide GalNAc was a weak competitor; it only slightly reduced the binding of TBCF10839 to the mucins. Conversely, compared to GalNAc the three core 2 *O*-glycans were moderate to strong competitors in a strain-, mucin- and oligosaccharide-dependent manner (*P* = 7x10^-4^, effect size 0.18). A strong competition was seen with the *O*-core 2 glycan to impede the adhesion of TBCF10839 to airway CF mucin from donor 2. Likewise, the *O*-core 2 extension type I prevented the adhesion of serotype 9 to CF mucin from donor 4 and the *O*-core 2 extension type II prevented the adhesion of serotype 9 to mucins from donors 2 and 4 and the adhesion of strain CF1-10 to mucin from donor 2 (**Table 4**). The tetra-antennary *N*-glycan reduced the adhesion of TBCF10839 to the CF mucin from donor 2 and the adhesion of CF1-10 to the CF mucin from donor 4 suggesting that the two CF isolates do not only recognize *O*-glycans, but also bind to the asparagine-linked complex-type oligosaccharides of *N*-glycosylproteins.

**Table 4:** Competition between soluble oligosaccharides and solid mucin for adhesion to P. aeruginosa*.

Relative fraction in percent of mucin-bound CFU in the presence and absence of oligosaccharide in PBS^#^																
Mucin	Glycan [µg/ml]	GalNAc			O-core 2			O-core 2, chain type I			O-core 2, chain type II			Tetra-antennary *N*-glycan		
		TBCF10839 CF1-10 IATS-09			TBCF10839 CF1-10 IATS-09			TBCF10839 CF1-10 IATS-09			TBCF10839 CF1-10 IATS-09			TBCF10839 CF1-10 IATS-09		
Mucin preparation from sputum of CF donor 2																
	5	100	260	110	80	40	70	40	40	20	70	70	50	60	150	40
	50	80	300	110	5	20	40	10	40	20	30	5	5	20	170	110
Mucin preparation from sputum of CF donor 4																
	5	50	120	300	70	120	40	50	100	20	50	100	80	60	20	300
	50	50	120	400	40	250	10	20	30	5	70	100	5	60	20	300
PSM																
	5	50	90	100	120	30	50	150	70	60	110	30	80	140	80	110
	50	60	120	90	60	100	70	110	20	50	80	20	70	40	60	70

*Values rounded to one digit are the mean of three technical replicates. Values larger than 100 indicate that the strain was growing in the presence of oligosaccharide.

^#^The fraction is given as the ratio of bound CFU to total CFU in percent in the presence of oligosaccharide normalized to the ratio of bound CFU to total CFU in percent in the absence of oligosaccharide.

## 4 Discussion

*P. aeruginosa* isolates from CF airways showed a strain-specific signature in their adhesion to submaxillary and CF airway mucins ranging from no or low to moderate and strong binding. Serial clonal isolates and even colony morphotypes from the same sputum sample were as heterogeneous in their affinity to mucin as representatives of other clones thus making ‘mucin binding’ one of the most variable intraclonal phenotypic traits of *P. aeruginosa* known to date. According to our knowledge, this is the first study that examined the adhesion of *P. aeruginosa* to respiratory mucin from CF donors. Previous work investigated bacterial adhesion to tracheobronchial mucins of non-CF donors (Vishwanath and Ramphal, 1984; Ramphal et al., 1991; Simpson et al., 1992; 1995; Carnoy et al., 1994; Ramphal and Arora, 2001). Thus, *P. aeruginosa* recognized a different spectrum of epitopes in the adhesion studies, because CF and non-CF mucins differ in their *O*-glycosylation profile. CF mucins present less core 3 and core 4 glycans than samples of non-CF origin (Bechtella et al., 2024). Sialylation is increased, while sulfation and fucosylation are decreased (Davril et al., 1999; Lamblin et al., 2001; Xia et al., 2005; Schulz et al., 2007; Hayes et al., 2012; Bechtella et al., 2024).

The strains of the PAO1 and PA14 lineages commonly used to study the biology of *P. aeruginosa*, were poor or no mucin binders. Strains PAO1 and PA14 have provided deep insights into key metabolic and regulatory pathways thought to be applicable to all *P. aeruginosa*, but in our case the sole use of the reference strains would have missed the prominent role of mucin binding for the lifestyle of *P. aeruginosa* in the CF lung and probably other mucus-covered habitats. Consistent with our observation, most previous studies on mucin binding from the 1980s and 1990s had not been performed with *P. aeruginosa* PAO1 or PA14, but with strains PAK and 1244 (Vishwanath and Ramphal, 1984; Ramphal et al., 1991; Simpson et al., 1992; 1995; Carnoy et al., 1994; Ramphal and Arora, 2001).

The majority of the tested isolates of the five clonal lineages (**Table 2**) adhered with similar affinity to wells coated with mucin and to uncoated wells of the same microtiter plate. This behavior should however not be qualified as ‘non-specific binding’. First, repeated exposures of strains cultivated from the same stock culture had qualitatively the same outcome of no, low, moderate or strong adherence to mucins over the whole study period of six years. The mucin adhesion assay was performed with planktonic bacteria during the late exponential growth phase, when strain-to-strain variation of density of cultures was minimal and the optical density was a robust measure of CFUs not distorted by bacterial clumping and exopolysaccharides (Sadovskaya, 2014). Second, the strength of adherence to polystyrene was also a strain-specific reproducible feature (**Table 2**). Hence, we would like to conclude that the adherence of *P. aeruginosa* to mucin (and to polystyrene) is a strain-specific inherited trait. Conversely, isolates of *Staphylococcus aureus*, the other major CF pathogen, did not attach to CF mucin (**Supplementary Table S2**) confirming the outcome of adherence studies with four Gram negative taxa (Vishwanath and Ramphal, 1984) that attachment to mucin is not a general bacterial property, but specific to *P. aeruginosa.*

The pioneering studies from the 1980s and 1990s had revealed that carbohydrate moieties are recognized by the bacterial adhesins. Consistent with this earlier work, strong binders of our collection adhered with similar affinity to the CF mucin preparation of donor 2 and its purified *O*-glycosylated PTS domains generated by cleavage of disulfide bridges with dithiothreitol and subsequent tryptic digestion of the non-glycosylated domains (data not shown).

Thanks to the improvements in mass spectrometry during the last few years, we now start to appreciate the diversity of *O*-glycans in airway mucins (Brockhausen et al., 2022). According to our experimental data, the majority of strains in our panel did not significantly discriminate between mucin and plastic or between individual CF mucin preparations. Thus, a broad supply of low affinity binding sites seems to be sufficient for *P. aeruginosa* to confer low or moderate non-selective binding to airway mucins. Conversely, a strain-specific gradient of affinity to the submaxillary mucins and the individual CF mucin preparations was noted for the few *P. aeruginosa* strains with strong mucin-binding capacity. Interestingly, dense glycosylation seems to be more important than a complex structure of the oligosaccharide. OSM having NeuAcα2–6GalNAcα1 and asialo-OSM having 6GalNAcα1 attached to almost all serine and threonine residues of their PTS domains (Konstantinidi et al., 2022), had not the lowest, but intermediate ranks for bacterial adhesion (**Supplementary Table S4**). However, more complex *O*-core 1 and *O-*core 2 oligosaccharides were recognized with higher affinity. The strongest binding was observed for PSM with its repertoire of *O*-core 1 oligosaccharides (Gerken et al., 2002) (**Supplementary Table S4**), irrespectively of whether the mucin was sialylated or non-sialylated. Since no model mucin with exclusive *O*-core 2 oligosaccharides was available, we performed competition experiments with benzylated *O*-core 2 compounds. The uncharged *O*-core 2 oligosaccharides efficiently prevented the adhesion to solid *O*-core 1 PSM in case of *P. aeruginosa* 1-10, but not in case of TBCF10839 showing the differential selectivity of these highest bacterial binders for oligosaccharides.

Outer membrane proteins and the flagellar cap protein FliD have been identified as mucin adhesins of the non-piliated PAK and 1244 strains (Carnoy et al., 1994; Ramphal et al., 1996; Arora et al., 1998). Likewise, mutants of the RNA polymerase σ^54^ factor RpoN showed a significant reduction of adherence to surfaces, epithelial cells and tracheobronchial mucins (Ramphal et al., 1991; Simpson et al., 1992; 1995). These proteins encoded by the core genome, however, were probably not involved in the mucin adhesion of our strongest binders 1-10 and TBCF10839. We have sequenced the genomes of the high mucin binder TBCF10839 and its clonal variant, the low mucin binder TBCF121838 (Klockgether et al., 2013; Wiehlmann et al., 2023). The two strains share an almost identical core genome. Besides four amino acid sequence variants, a large deletion in the *pilQ* gene of TBCF10839 is the major difference between the two genomes. As a consequence of this single loss-of-function mutation, the two clonal variants of the TB clone differ in the expression of hundreds of genes of their core genome which probably led to the divergent traits in virulence, motility and adhesion to respiratory and submaxillary mucins (Klockgether et al., 2013). Patient 2 had been critically ill during carriage of the TB clone with high mucin binders such as strain TBCF10839 (Tümmler et al., 1991; Klockgether et al., 2013), but he recovered after the TB clone had been replaced by the chronic carriage of the PA14 clone (D421) with its poor mucin binders (**Tables 2**, **3**).

We also have examined the microevolution of the 1BAE clone in patient 1 by whole genome sequencing of serial isolates (Klockgether et al., 2018). During the first two years of colonization with *P. aeruginosa* (see **Table 2**) six missense variants and two loss-of-function frameshifts emerged in the core genome, the latter in *xcpR* encoding the traffic ATPase of the type II secretion system and in *rpoN* encoding the σ^54^ factor. *RpoN* mutants in strains PAK and 1244 have been reported to be practically non-adhesive to mucin (Ramphal et al., 1991; Simpson et al., 1992; 1995). At first glance, it is surprising that the *rpoN* mutant CF1-10 showed stronger adhesion to mucins than its *rpoN* – proficient 1BAE ancestor (**Table 2**). However, the inspection of the accessory genomes of ancestor and progeny provides a clue. Only about 400 of the more than 1,000 ORFs could be annotated by homology with an entry in the GenBank and ENA databases. Of the 400 annotated ORFs, the serial isolates shared just 145 annotated ORFs. Based on these in silico findings, we hypothesize that the accessory genome encodes the mucin adhesins of the 1BAE clone. Preferentially the ‘dark matter’ of ‘orphan ORFs’ lacking currently any homologous gene in the databases may comprise yet undescribed adhesins. Horizontal gene transfer continuously modifies the intraclonal genetic repertoire. Mobile genetic elements such as the Integrative and Conjugative Elements (ICE) are proficient to exchange genes even across species barriers in the microbial communities of the airways of the CF host (Delavat et al., 2017) and the evolutionary race between phage and bacterium speeds up the turnover of genes in the accessory genome (Tiamani et al., 2022). Thus, the clonal community can diversify in their affinity and specificity of mucin binding as seen for the three 1BAE colony morphotypes CF1-8, CF1-9 and CF1-10 from the same sputum sample. Their variable adhesion to mucin assigned to a differential composition of the accessory genome is in line with their differential phenotype of phage susceptibility pattern and pyocin type both of which exclusively specified by the accessory genome (data not shown). In summary, the two cases of strong mucin binders teach us that the adhesion to respiratory mucin is a rapidly evolving trait in *P. aeruginosa* that contributes to intraclonal diversification. The higher the affinity to mucin, the more specific are the epitopes recognized by the *P. aeruginosa* strain.

Our study has limitations. First, we adapted the protocol by Vishwanath and Ramphal (1984) to determine the attachment of *P. aeruginosa* to mucin in 96-well plates. Despite all precautions to work as reproducibly as possible, both the inoculum determined by OD and the outcome determined by CFU were subject to large biological variation (cf. the primary CFU data shown in the Excel table in the supplement). Hence, the experimental data shown in **Tables 2**–**4** should be considered as semi-quantitative in nature. Despite this unavoidable scatter of the biological replicates, the repeated experiments performed from the stock cultures over a period of six years worked reproducible and robust in terms of the categorization of the strains in no, low, moderate, strong and very strong binders and their pairwise classification (**Supplementary Table S4**).

Second, due to the lack of adequate instruments, we could not determine the profile of *O*-glycans in our sputum-derived CF mucin samples. To compensate this imperfection, we complemented our study with oligosaccharide competition experiments and the inclusion of submaxillary mucins of known differential complexity of their *O-*glycans. Mass spectrometry analysis of mucin-type *O*-glycans has made dramatic progress in recent years, particularly ion mobility - tandem mass spectrometry as the most recent addition, but the reader may note that the published in depth analyses have mainly been performed on singular samples of submaxillary or intestinal mucins. Respiratory secretions are mixtures of MUC5B and MUC5AC with or without membrane-bound mucins as minor components (Bechtella et al., 2024). According to our knowledge, the properties of native human respiratory mucins have always been examined in mixtures. Past studies from the pre-cloning era on purified preparations investigated isolated domains, but not the native polydisperse multi-domain *O*-glycoprotein. Having the rationale that our mucin binding studies should mimic the real world of the adhesion of *P. aeruginosa* in CF lungs, we decided to maintain all native mucin components from the CF sputum sample in our preparation. Hence, we followed Snyder’s lengthy protocol from 1982 to remove ions, metabolites and non-mucin proteins by multiple centrifugation, dialysis and lyophilization steps without any electrophoretic or chromatographic separation by molecular weight. To our satisfaction, PAGE and gel elution chromatography did not detect any low molecular weight proteins in the final CF mucin lyophilizates. Their monosaccharide composition matched with those of MS studies on CF mucins. Thus, our study on the adhesion of *P. aeruginosa* to CF mucins should reflect the scenario of *P. aeruginosa* in the conducting airways of people with CF as close as possible, the bacterium being embedded in microcolonies made up of CF mucins as the major solid constituent.

In our study we commonly noted disparate mucin affinity profiles among the intraclonal morphotypes of a sputum sample indicating that the CF lung is colonized with a heterogeneous community of low to high affinity mucin binders. The extensive spatiotemporal variation of the adhesion to mucin probably reflects the rapid adaptation of *P. aeruginosa* to a permanently changing habitat. In line with this conjecture, all strong mucin binders were isolated during a pulmonary exacerbation that required hospitalization and antipseudomonal chemotherapy. Despite this interesting association between the bacterial adhesion phenotype and the disease status of its CF host, we should be cautious when extrapolating our results to the real-world scenario in the CF lung. The standardization of the test comes at the cost of inherent limitations. Adhesion is studied with planktonic bacteria during late exponential growth, morphotypes are excluded that cannot be reliably quantified by optical density or plating and the mucins were isolated from late stage lung disease when CF patients still produce large quantities of sputum. Our test moreover ignores important aspects of the life of *P. aeruginosa* in CF airways such as the co-colonization with other taxa of the microbiome, the spatially heterogeneous habitat of remodeled and/or inflamed airways at the macroscopic scale and the gradient of oxygen and metabolites within the microcolony at the microscopic scale.

With the exception of the few strong mucin binders (**Supplementary Table S5**), the CF airway isolates like the reference strains of non-CF origin bound to mucins with comparable affinity irrespective of *O*-glycan complexity. Thus, mucolytics that target these high-density low affinity mucin – bacterium interactions should be the prime target of therapeutic intervention. Efficient mucolytics are not only beneficial for people with CF, but also for patients with other muco-obstructive diseases such as bronchiectasis and advanced COPD. Carbocysteine and *N*-acetylcysteine, focused on mucin disulfide bond reduction, have been used for many years to treat muco-obstructive lung disease with evidence of only limited efficacy (Mall et al., 2018). However, meanwhile some promising compounds have been developed. A low molecular weight 12-15-mer alginate oligosaccharide (OligoG CF-5/20) derived from plant biopolymers, induced alterations in mucin surface charge and porosity of the three-dimensional mucin networks in CF sputum (Pritchard et al., 2016). A phase 2b study, however, did not indicate a significant benefit of the inhalation of OligoG-dry powder compared to placebo (van Koningsbruggen-Rietschel et al., 2020). P3001, a mucin-reducing agent (Ehre et al., 2019), and MUC-031, a thiol-modified carbohydrate compound (Addante et al., 2023), were tested in CF sputum and in mice with muco-obstructive lung disease (βENaC-Tg mice). Both P3001 and MUC-031 were more effective than rhDNase and *N*-acetylcysteine to reduce the viscoelasticity of CF sputum *in vitro*. Moreover, both compounds significantly decreased lung mucus burden, lessened inflammation and improved survival in the mouse model *in vivo*. A thiol-containing, sulfated dendritic polyglycerol (dPGS-SH) is the most recent addition to the portfolio of mucolytics (Arenhoevel et al., 2024). The dPGS-SH polymer demonstrated mucolytic activity in CF sputa and cleaved MUC5AC and MUC5B more effectively than *N*-acetylcysteine. These novel thiol-containing compounds are worthwhile to be tested in clinical trials whether they are more efficient than *N*-acetylcysteine in the treatment of muco-obstructive lung disease.

## Data Availability

The datasets presented in this study can be found in online repositories. The names of the repository/repositories and accession number(s) can be found below: https://www.ebi.ac.uk/ena, ERP001300; https://www.ncbi.nlm.nih.gov/genbank/, PRJNA975170.

## Supplementary material

Supplement 1 WORD document.

Supplement 2 Excel document.

Supplementary Table 6 Adhesion of serial *P. aeruginosa* isolates to CF respiratory mucin preparations: Mean of primary CFU data of biological replicates.
