# Supplementary material for: Mucin adhesion of serial cystic fibrosis airways Pseudomonas aeruginosa isolates
Source: Front Cell Infect Microbiol. 2024 Aug 22;14:1448104. doi: 10.3389/fcimb.2024.1448104 (PMC11374773; doi:10.3389/fcimb.2024.1448104)
Supplement: Supplement 1 — WORD document. [file Table1.docx]

Supplement

Supplementary Table S1. Optimization of the mucin adhesion assay.

1. Recovery of *P. aeruginosa* TBCF121838 bound to a polystyrene well with detergent Triton X-100. Wells of the microplate were inoculated with 100 µL of a tenfold-dilution of a bacterial suspension of OD_600_= 0.6 (≈ 10^7^ bacteria) and incubated for 30 min at 37°C. By the end of the incubation period, the fluid was removed. Then the respective concentration of Triton X-100 in PBS was added to each well and incubated for 30 min at room temperature to release bound bacteria to be quantified by CFU. Data is the mean of three technical replicates (range of SD: 20% – 30% of individual means) from one microplate.

Triton X-100 concentration [% w/v] Recovered bacteria / well

0 1.3 x 10^5^

0.25 2.4 x 10^5^

0.5 3.0 x 10^5^

1.0 3.2 x 10^5^

2.0 2.2 x 10^5^

1. Recovery of *P. aeruginosa* TBCF121838 bound to a polystyrene well with detergent Triton X100: Variation of exposure time to 1.0 % [w/v] Triton X-100. Data is the mean of three technical replicates (range of SD: 23% – 30% of individual means) from one microplate.

Exposure time [min] to 1% Triton X-100 Recovered bacteria / well

5 1.1 x 10^5^

15 5.1 x 10^5^

30 4.4 x 10^5^

45 3.3 x 10^5^

60 0.9 x 10^5^

1. Removal of non-adhering bacteria by washing with PBS: Variation of the number of washing steps with PBS. Uncoated wells of the microtiter plate were exposed for 30 minutes with an inoculum of *P. aeruginosa* PAO1 or IATS O9 bacteria. Non-adhering bacteria were then removed by 5, 10 or 20 washing steps with PBS. Next, the residual adhering bacteria were released by exposure to 1% Triton X-100 for 30 minutes and the recovered bacteria were quantified by CFU. The data demonstrates that an adhesion to mucin of less than 1% cannot be discriminated from non-specific binding.

Inoculum [CFUs] 336x10^3^  359x10^3^  108x10^3^ 122x10^3^

Residual bacteria after

5 washing steps 13x10^2^ 19x10^2^  5x10^2^  112x10^2^

10 washing steps 17x10^2^  15x10^2^  5x10^2^  60x10^2^

20 washing steps 15x10^2^  14x10^2^  3x10^2^  47x10^2^

Inoculum [CFUs] 47x10^5^  59x10^5^  105x10^5^ 100x10^5^

Residual bacteria after

5 washing steps 409x10^2^ 141x10^2^  210x10^2^  290x10^2^

10 washing steps 55x10^2^  40x10^2^  112x10^2^  149x10^2^

20 washing steps 5x10^2^  20x10^2^  34x10^2^  35x10^2^

1. Adhesion of the clonal variants *P. aeruginosa* TBCF121838 and *P. aeruginosa* TBCF10839 to CF mucin 4: Variation of mucin concentration. Wells were coated with 200 µL of the respective concentration of CF mucin 4 in PBS. Mucin-coated wells were inoculated in triplicate with 100 µL of a tenfold-dilution of a bacterial suspension of OD_600_ = 0.6 (≈ 10^7^ bacteria). After 30 min incubation the fluid was removed from the wells. After washing the wells 10 times with 250 µl PBS per well, 300 µl 1% [w/v] Triton X 100 in PBS was added to each well and incubated for 30 min at room temperature to release mucin and bound bacteria. Thereafter the released bacteria were quantified by CFUs that were determined by plating tenfold dilutions of 100 µl aliquots of the Triton X100 suspension. Data is the mean of three technical replicates (range of SD: 25% – 30% of individual means) from one microplate.

Concentration of CF mucin C Bacteria bound to the mucin-coated well

[µg / ml] TBCF121838 TBCF10839

0 2.4 x 10^4^  1.5 x 10^4^

0.01 5.6 x 10^4^ 8.5 x 10^4^

0.05 7.4 x 10^4^  1.1 x 10^5^

0.1 6.8 x 10^4^  9.0 x 10^4^

0.5 9.2 x 10^4^  2.2 x 10^5^

1 4.1 x 10^5^  4.3 x 10^5^

5 4.8 x 10^5^  3.8 x 10^6^

10 4.1 x 10^5^  3.3 x 10^6^

50 2.9 x 10^5^  2.0 x 10^6^

100 2.3 x 10^5^  2.2 x 10^6^

According to this data shown in A, B, C and D, the mucin adhesion assay was standardized to a coating with 200 µL mucin (10 µg/ml PBS) and to 30 min exposure with 1% Triton X-100 to release mucin and bacteria from the polystyrene well.

Supplementary Table S2. Adhesion of *Staphylococcus aureus* isolates from bronchial secretions of hospitalized patients to CF mucin 4 [10 µg/ml PBS]. Mucin-coated wells were inoculated in triplicate with 100 µL of the bacterial suspension. After 30 min incubation the fluid was removed from the wells. After washing the wells 10 times with 250 µl PBS per well, 300 µl 1% [w/v] Triton X 100 in PBS was added to each well and incubated for 30 min at room temperature to release mucin and bound bacteria. Thereafter the released bacteria were quantified by CFUs.

Bacterial strain Bacterial inoculum [CFU] bound bacteria [CFU]* % bound of inoculum

*S. aureus* 1 1 x 10^8^ 1.4 x 10^3^  0.001

*S. aureus* 2 1 x 10^7^ 6.6 x 10^3^  0.07

*S. aureus* 3 4 x 10^7^ 4.6 x 10^3^  0.01

*S. aureus* 4 4 x 10^7^ 1.4 x 10^4^  0.04

*S. aureus* 5 6 x 10^7^  3.9 x 10^3^  0.007

TBCF121838 2 x 10^6^  1.1 x 10^5^  6

TBCF10839 2 x 10^7^  4.5 x 10^6^  22

*mean of three technical replicates

Supplementary Table S3. Mann-Whitney U Rank tests of the binding of serial *P. aeruginosa* isolates to polystyrene and CF mucin preparations whether the affinity of the mucin – bacterium interaction is more driven by the chemical nature of the mucin/polystyrene (‘receptor’) or by the repertoire of adhesins of the bacterial strain or bacterial clone. Datasets A and B were compiled from the adhesion data listed in Table 2. The approach is explained in section 2.6 ‘Statistics’. Dataset A lists all pairwise comparisons of the ratio of bound CFU to total CFU to one target for all colony morphotypes retrieved from one sputum sample (I) or one genotype (II), compiled for all 4 targets. Dataset B lists all pairwise comparisons of the ratio of bound CFU to total CFU of one colony morphotype to four targets (polystyrene and the mucin preparations from CF patients 3, 4, 5), compiled for all colony morphotypes retrieved from one sputum sample (I) or one genotype (II).* The ranks of the individual compound fractions of the combined datasets A and B were then assessed by Mann Whitney test.

Clone type colonization time [yrs] Z score *P* value

1. Three or more isolates from one time point

1BAE 0.7 1.933 0.0268

1BAE 1.3 2.277 0.0266

D421 n.s.

6D92 4.3 3.248 0.00058

E84A 5.3 4.551 < 0.00001

0C2E 10.8 6.094 < 0.00001

1. All isolates from a clonal lineage

1BAE 2.342 0.00964

D421 2.055 0.0197

6D92 3.282 0.00058

E84A 4.253 < 0.00001

0C2E 6.847 < 0.00001

*Ranks were assigned to the absolute values of the fraction

[ (bound cfu (1) / total cfu (1)) : (bound cfu (2) / total cfu (2)) ≥ 1 ], i.e., the fold difference between two ratios

Supplementary Table S4. Post-Hoc Dunn’s test of pairwise comparison of the adhesion of *P. aeruginosa* strains to the seven CF mucin preparations, five submaxillary mucins and polystyrene using a Bonferroni corrected alpha of 0.00055.

Uncorrected *P* values of pairwise comparisons by Mann – Whitney rank tests*

Bacterial strain CF1-7 CF1-8 CF1-10 CF2-7 CF2-8 CF3-7 CF4-4 CF5-8 CF5-9 PAO1 IATS04 IATS06 IATS09

TBCF10839 ns ns ns **0 0** ns **7x10^-8^** 4x10^-3^ ns **5x10^-9^ 5x10^-4^ 7x10^-9^ 5x10^-6^**

CF1-7 ns 1x10^-3^ **6x10^-12^ 1x10^-10^** ns **5x10^-4^** ns ns **9x10^-5^** ns **1x10^-4^** 7x10^-3^

CF1-8 ns **0 3x10^-5^** ns **7x10^-7^** 2x10^-3^ ns **6x10^-8^** 2x10^-3^ **9x10^-8^ 3x10^-5^**

CF1-10 **0 0** 3x10^-3^ **3x10^-11^ 1x10^-6^** 4x10^-3^ **1x10^-12^ 2x10^-6^ 2x10^-12^ 5x10^-9^**

CF2-7 ns **2x10^-12^** 7x10^-4^ **2x10^-7^ 7x10^-13^** 3x10^-3^ **1x10^-7^** 3x10^-3^ **3x10^-5^**

CF2-8 **4x10^-11^** 3x10^-3^ **2x10^-6^ 2x10^-11^** ns **1x10^-6^** ns **2x10^-4^**

CF3-7 **3x10^-4^** ns ns **4x10^-5^** ns **6x10^-5^** 5x10^-3^

CF4-4 ns **2x10^-4^** ns ns ns ns

CF5-8 ns ns ns ns ns

CF5-9 **2x10^-5^** ns **3x10^-5^** 3x10^-3^

PAO1 ns ns ns

IATS 04 ns ns

IATS 06 ns

*significant *P* values after Bonferroni correction are given in bold font; non-significant *P* values < 0.01 are given in normal font; ns, not significant *P* values > 0.01

Supplementary Table S5. Selectivity of the adherence to mucins of the most strongly binding *P. aeruginosa* strains

*P. aeruginosa* strain Mucins*

TBCF10839 PSM ≈ a-PSM > 2 ≈ 3 ≈ 7 ≈ a-OSM ≈ BSM > 5 ≈ OSM > 6 > 4

CF1-7 (1BAE) 2 ≈ a-PSM > 4 ≈ BSM > 3 ≈ 5 ≈ 7 ≈ OSM ≈ a-OSM > 6 > PSM

CF1-8 (1BAE) PSM ≈ a-PSM > 5 ≈ 6 ≈ a-OSM > 2 ≈ 4 ≈ OSM ≈ BSM > 3 ≈ 7

CF1-10 (1BAE) a-PSM > 2 ≈ BSM > 5 ≈ OSM ≈ a-OSM ≈ PSM > 6 > 3 ≈ 4 ≈ 7

CF3-7 (6D92) 5 > 3 ≈ 6 > OSM ≈ PSM > 4 ≈ 7 ≈ a-PSM > 2 ≈ a-OSM ≈ BSM

CF5-9 (0C2E) 2 > PSM ≈ a-PSM > 4 ≈ 6 ≈ BSM ≈ a-OSM > 7 ≈ OSM > 3 ≈ 5

*Mucins are ranked from strong to weak bacterial adherence; ≈ similar affinity, same rank
